# *Eucalyptus* Pollen Allergy and Asthma in Children: A Cross-Sectional Study in South-East Queensland, Australia

**DOI:** 10.1371/journal.pone.0126506

**Published:** 2015-05-04

**Authors:** Jane E. M. Gibbs

**Affiliations:** School of Medicine, Griffith University, Southport, Queensland, Australia; National Heart and Lung institute, UNITED KINGDOM

## Abstract

**Objectives:**

To investigate *Eucalyptus* (gum tree) pollen allergy in children in relation to geography, particularly vegetation, and its relationship to asthma.

**Methods:**

Males (n = 180) and females (n = 200) aged 9 to 14 participated. Some were healthy (asymptomatic), some had asthma, and some had other symptoms associated with atopy. School students were from three urban coastal schools and one school from a nearby semi-rural elevated area (range) near Brisbane, Australia. Coastal and range locations featured different distributions of Myrtaceae family vegetation (including *Eucalyptus*, *Melaleuca*, *Leptospermum* species). Skin prick test (SPT) responses to 15 commercial allergens were compared. As well, responses from coast versus range groups, and ‘asthma’ (n = 97) versus ‘healthy’ status (n = 107) groups, were compared.

**Results:**

SPT responses (≥3mm wheal diameter) indicate that children with asthma are 31.1 times more likely to be allergic to *Eucalyptus* pollen extract (OR: 31.1; 95%CI 4.1- 235.7) compared to healthy children. Dust mite (p = .018), Eucalyptus (p = .046) and cockroach (p = .047) allergen SPT responses (wheals ≥3mm) were significantly greater in participants located on the coast versus range as determined by Fisher’s Exact Test (α .05). For each location, percentage of positive responses (wheals ≥3mm) was greatest for ‘dust mite’ (30.9%-46%), ‘cockroach’ (18.1% -35%) and ‘Bermuda grass’ (10.6%-19.4%).

**Conclusions:**

The results support the hypothesis that proximity to Myrtaceae vegetation is related to positive SPT response and that *Eucalyptus* is an important allergen for children with asthma. Substantial response to olive allergen, in the absence of olive trees, suggests that the response may be driven by substances in other plants, perhaps *Melaleuca quinquenervia*, which abounds in coastal areas.

**Implications:**

Response to *Eucalyptus* allergen indicates that changes in gardening practice in schools and public areas may be appropriate. The findings pose validity questions regarding the use of some commercial allergens due to cross-reactive responses and the sources of those responses.

## Introduction

Asthma prevalence in Australia is high compared to other countries and Queensland’s estimate of the ‘current asthma’ rate of 11.8% exceeds the national average of 9.9%, about two million people nationwide[1].

For more than 40 years in Brisbane, Queensland’s capital, seasonal asthma peaks in autumn with a smaller one in spring, have been identified [2].Those peaks are not well understood nor is the high prevalence of asthma in Australia and New Zealand generally[3]. Inside homes, avoidance of house-dust mite and parental smoking, in conjunction with breast-feeding and increased ingestion of omega-3 fatty acids, has shown promising reduction in wheeze in children [4]. The association between asthma and cockroach allergy has also been established [5] but has only limited explanatory value for ‘current asthma’ [6]. Outside the home fungal spores, pollens [7] and anthropogenic pollutants [8] are associated with respiratory symptoms but adequate explanatory variables are elusive.

**Fig 1:**
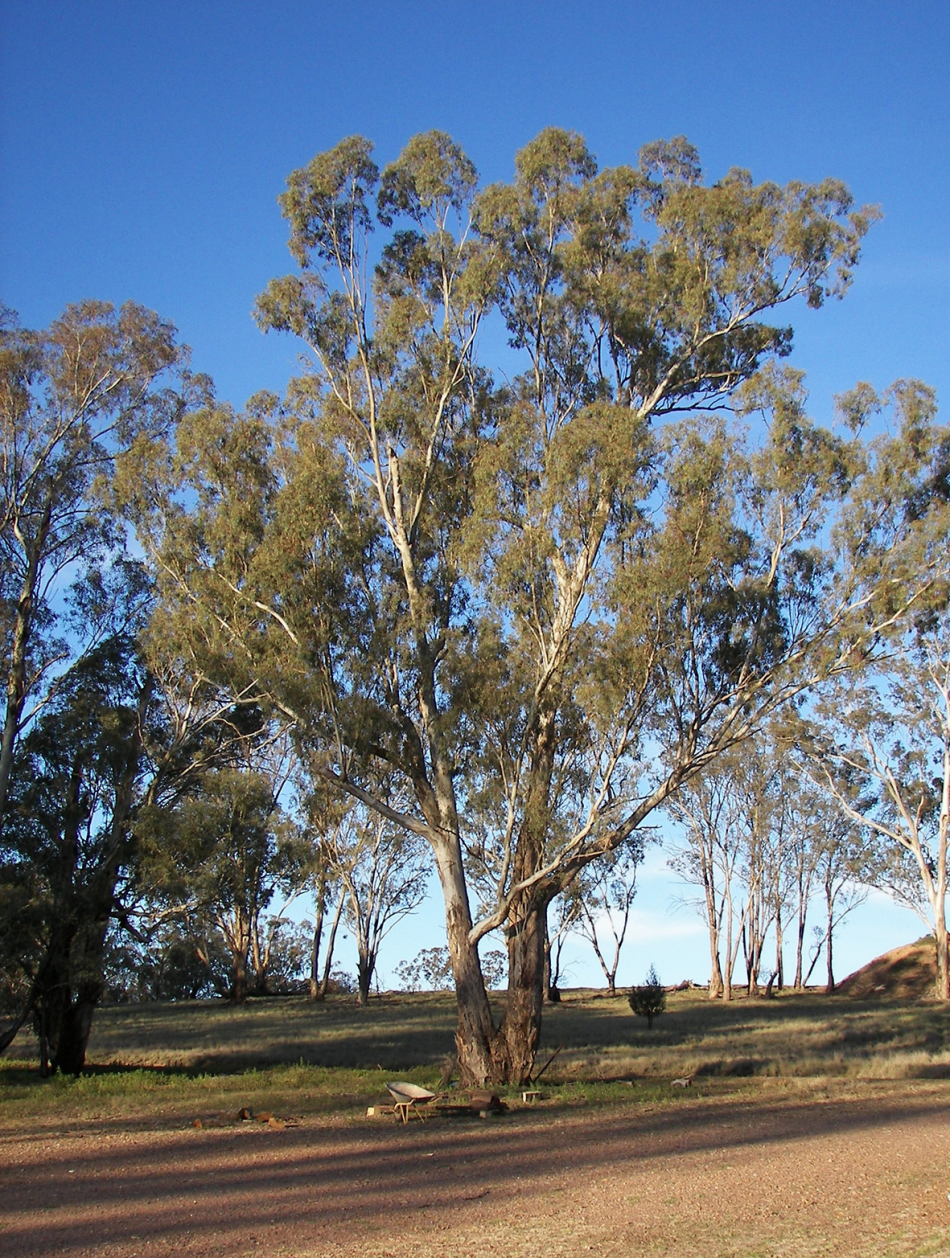
*Eucalyptus* tree.

Research into the role of Australian native vegetation, much of which belongs to the Myrtaceae family, is lacking. The *Eucalyptus* or ‘gum’ tree (Fig 1) is a prominent member.

This investigation seeks to enhance the currently poor knowledge base regarding allergy and plants from Australasia, and challenge the assumptions that deter scrutiny of common Australasian plants as possible respiratory symptom triggers.

### Absent evidence

**Fig 2:**
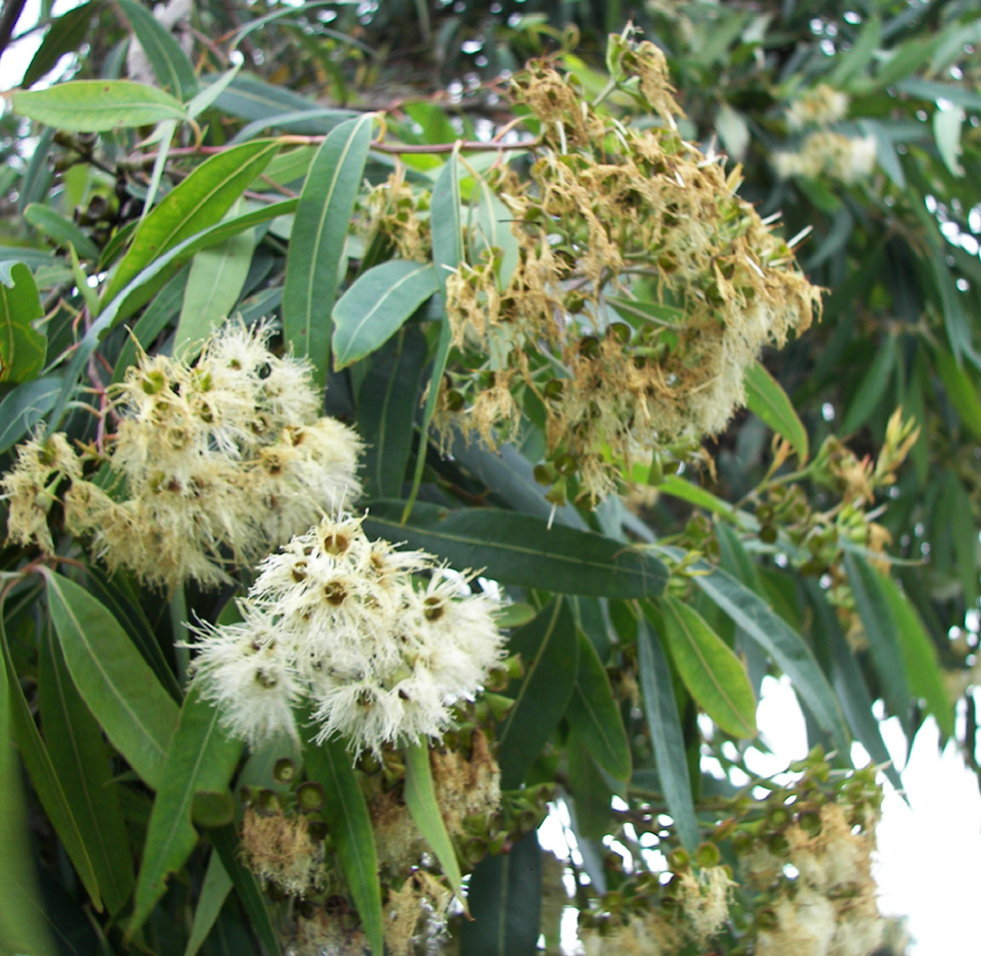
*Eucalyptus* flowers.

Myrtaceae plants have been long been regarded as safe garden alternatives to wind pollinated (anemophilous) plants for those with asthma and allergies. This tradition continues[9] despite a paucity of evidence to support that position. Instead of wind, insect (entomophilous) and animal pollinated (zoophilous) plants rely upon the emission of volatile chemicals, largely terpenoids, to deliver specific scent messages to preferred pollinators, often from the brush-like flowers (Fig 2). Some of these substances have been shown to be sensitizing[10–12].

Lewis and Vinay[13], identify the Myrtaceae family as producing problematic ‘inhalant effects’ in Hawaii. Stablein et al [14] investigated *Melaleuca quinquenervia*, introduced to Florida from Australia, and concluded that reports of allergic effects from *M*.*Quinquenervia* (cream bottlebrush) were probably due to its cross-reactivity with Bahia grass pollen.

### Floral frames for the asthma picture

**Fig 3:**
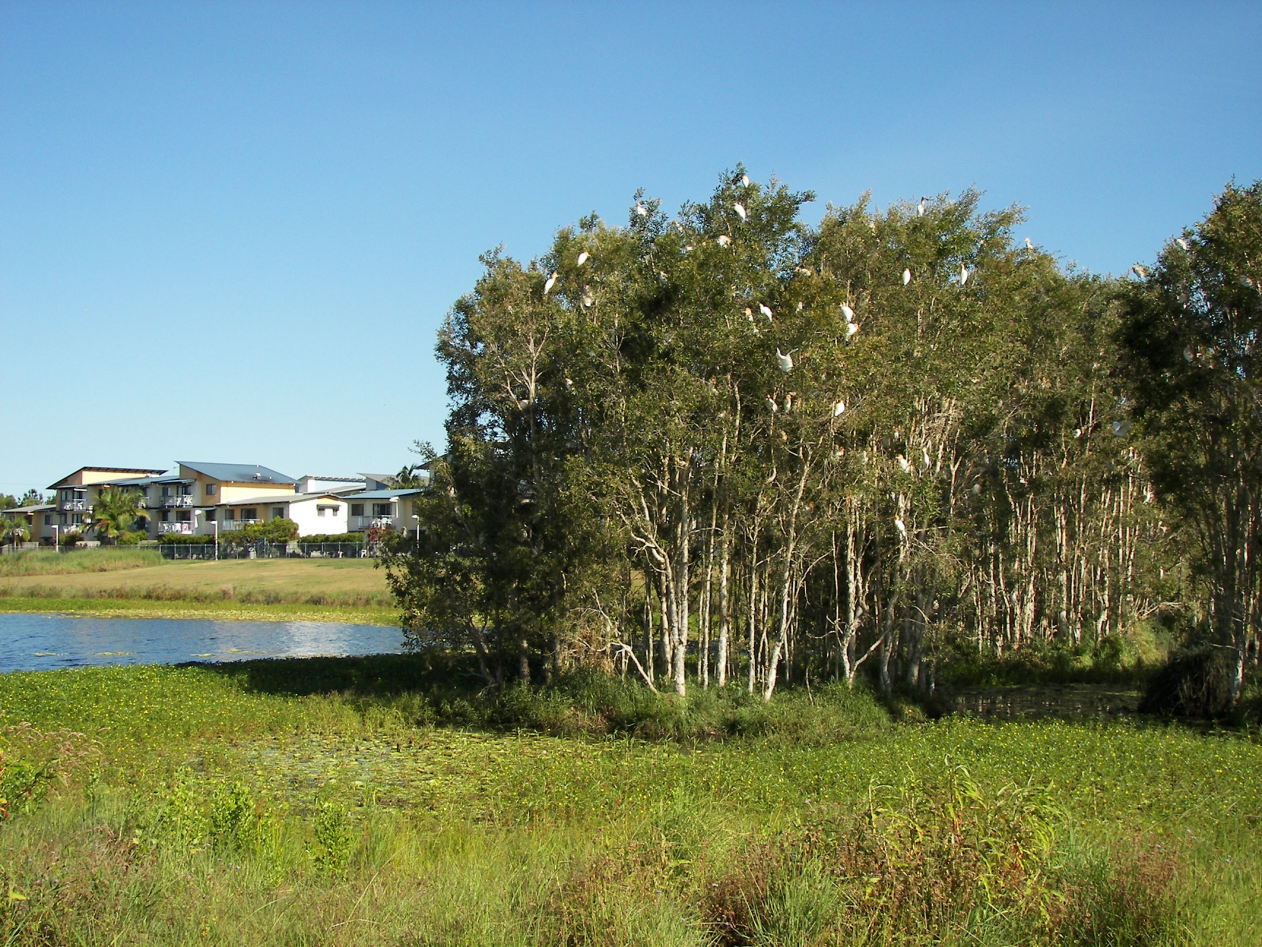
*Melaleuca quinquenervia* wetland and coastal residences.

**Fig 4:**
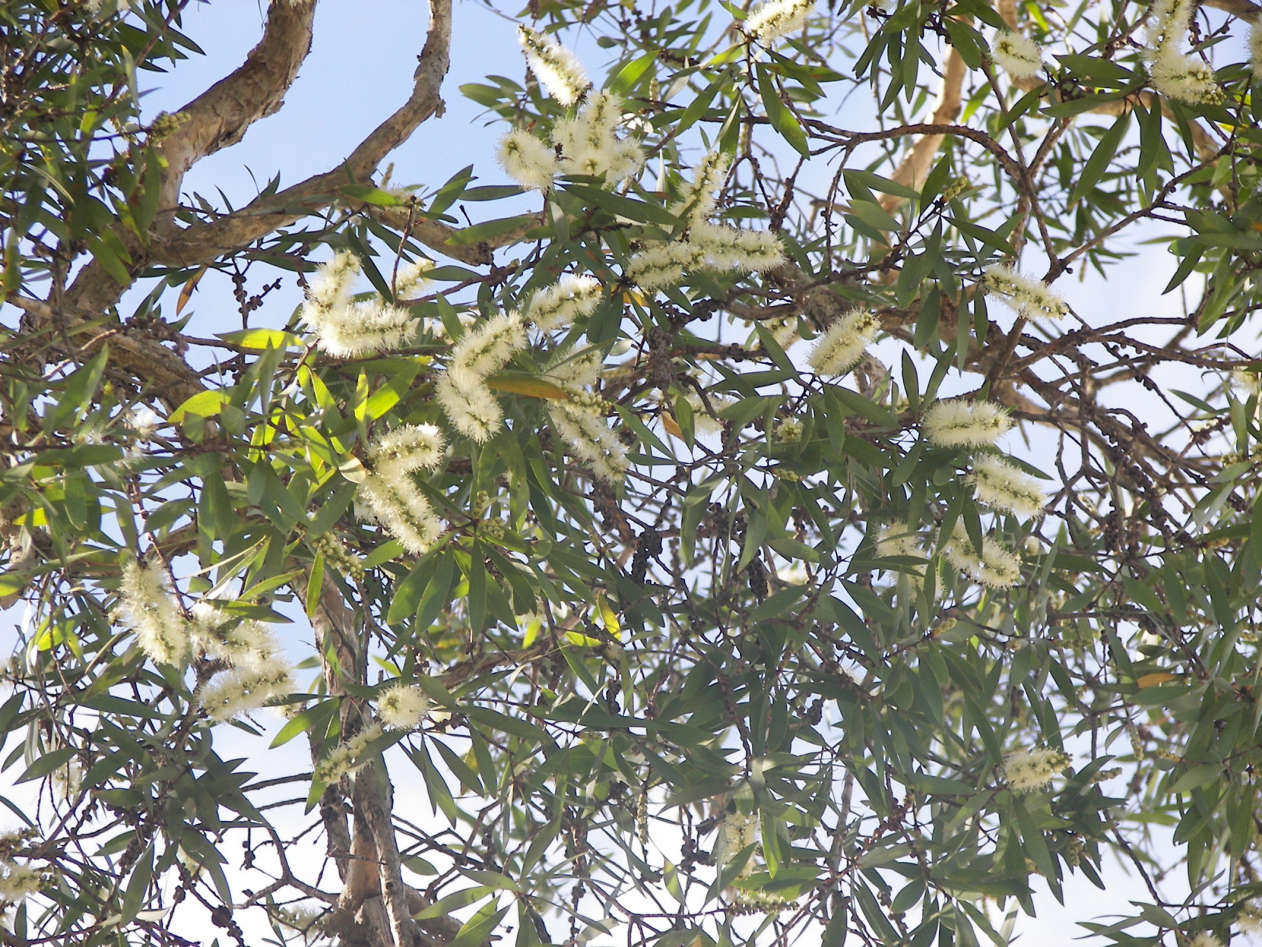
*Melaleuca quinquenervia* flowers.

Northern NSW and South-Eastern Queensland are home to vast numbers of *Melaleuca quinquenervia* [15] (Fig 3) which starts flowering (Fig 4) in late summer, peaks in autumn, and finishes in early winter. Flowering coincides with the asthma peak in S.E. Queensland. *Casuarina* (she-oak or Australian Pine) species are common and can flower from autumn to spring. The majority of prevalent *Eucalyptus* and *Acacia* (wattle) species flower in winter to spring in this region although some flower at other times. *Leptospermum* (tea-tree) species flower mostly in spring. These plants feature on the populated flat coastal strip that separates the Great Dividing Range from the Pacific Ocean.

Travelling inland, as the landform elevates, the vegetation is much more varied. Crops, orchards, rich grazing pastures, mixed and commercial forests and woodlands feature. *Eucalyptus*, *Callitris*, *Casuarina*, *Pinus*, *and Acacia* species are often seen.

Brisbane is a sprawling city of over 2 million people. It has grown simultaneously with the popularity of Australian native plants as garden specimens. What began as increased interest in the 1960s became a ‘boom’ by the 1980s[16]and replaced a formerly European garden tradition. *Eucalyptus*, *Melaleuca*, *Callistemon* and *Leptospermum* species can be seen extensively planted along streets, in gardens, around schools and in most public green spaces.

**Fig 5:**
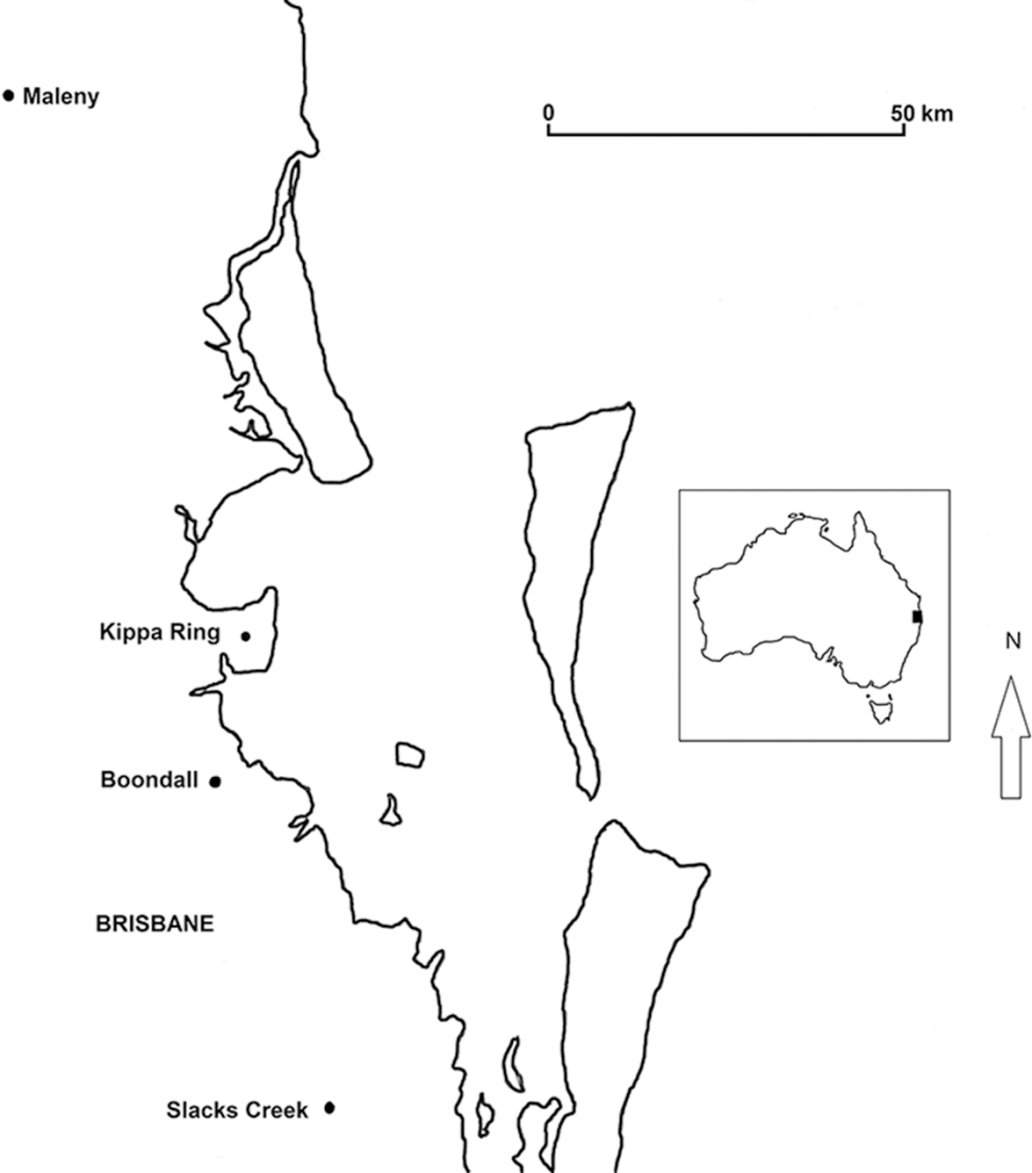
Schools locations.

This research investigates allergen sensitization patterns that may differ according to geography, specifically, the vegetation. Child participants from three coastal schools (average elevation 14m), from greater Brisbane, and a Blackall Range school (elevation 416m) from the Sunshine Coast region (Fig 5) were enlisted. *Melaleuca quinquenervia* only grows extensively on the coastal wetlands and does not grow naturally on the elevated range area selected. Proximity to large tracts of these Myrtaceae trees determined selection of the coastal schools.

The coastal schools are suburban consisting of mostly detached homes set on blocks of average 600sqm. The Range school in Maleny is part of a rural tourist town of approximately 4000 population where most of the children also live in detached homes on average blocks in town, and some on small acreage.

### The hypothesis

It was hypothesised that any response to *Eucalyptus* flower pollen allergen, representative of Myrtaceae family plants, would be similar in the three coastal schools and differ from the Range school because of proximity to Myrtaceae family vegetation, either natural or purposefully planted.

*Acacia* and *Casuarina* (Australian Pine) are found similarly in both areas and were not expected to differ between coastal and range schools.

**Fig 6:**
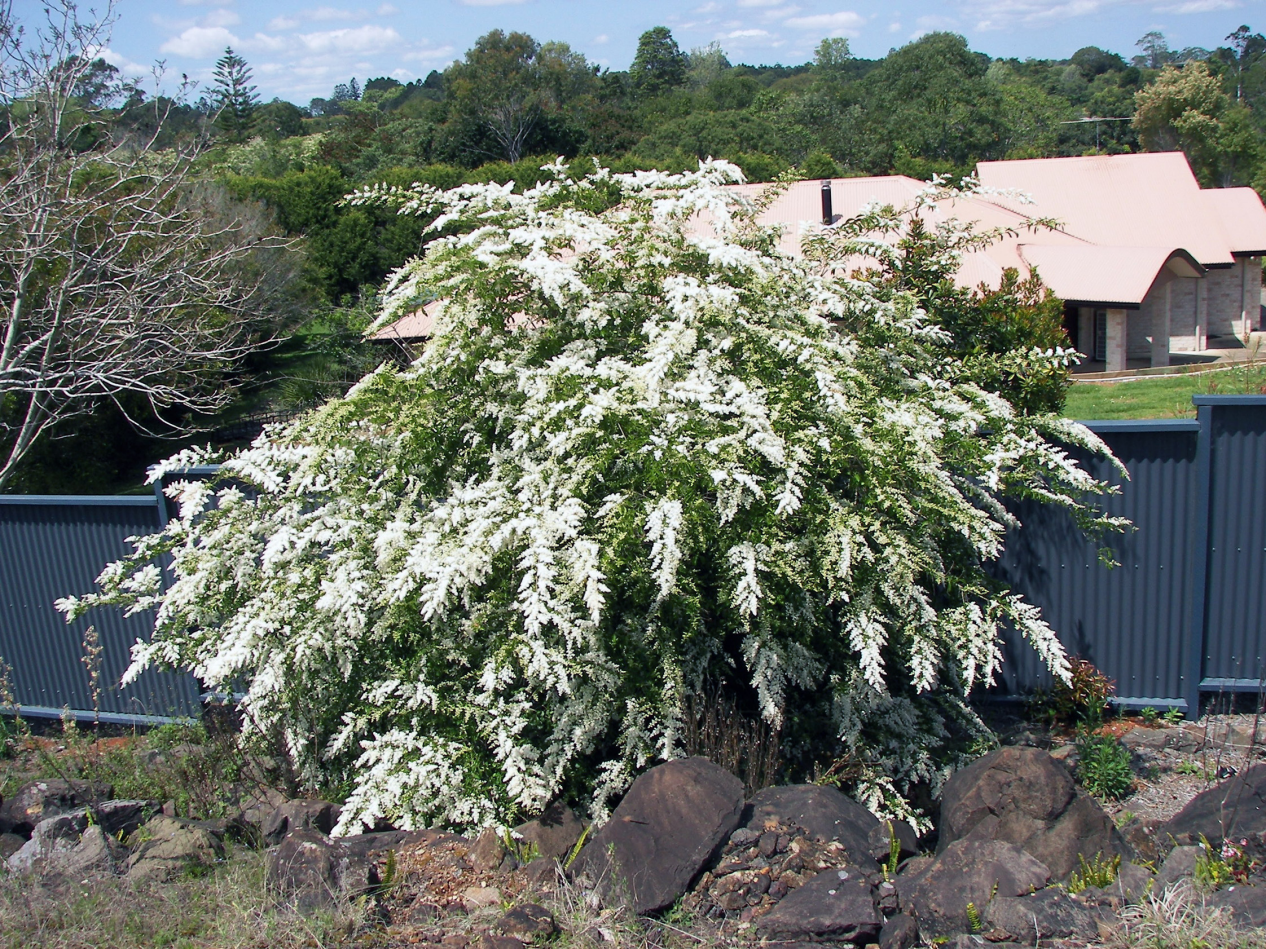
Small-leaved privet, *Ligustrum sinense*.

Response to privet was also of interest. It infests the Maleny area but not obviously the urban coastal areas. Olive (*Olea*), which cross-reacts with privet[17], was used here due to unavailability of privet allergen. Olives require dry summers, not wet ones that feature in Queensland and are not apparent in the area in question. Linalool, a terpenoid, from olive is also found in *Melaleuca* [18] and privet (*Ligustrum*) [19](Fig 6) and is the familiar honey scent that attracts bees. Will children from coastal schools respond to olive allergen perhaps because coastal Myrtaceae trees contain similar substances to olive or privet?

## Method

### Ethics Statement

Ethics approval was sought and obtained from the Griffith University Human Research Ethics Committee. Informed written consent was obtained from parents or guardians and children before children were tested.

### Recruitment of participants

Following ethics approval, children were recruited from four state schools of the same mid-range socio-economic band (designated by the Queensland Education Department): Kippa Ring (KR), Maleny (M), Boondall (B) and Slacks Creek (SC).

Volunteers from grades 4 to7 with and without respiratory and allergic symptoms were requested via the school newsletter. Parents or guardians were promised copies of skin test results. Children were given an ISAAC QUESTIONNAIRE (International Study of Asthma and Allergies in Childhood), a personal details form and an “informed consent” form for completion by carers. Children were tested, each school in turn, in one session each, one week apart in September/October 1998. The same staff administered allergens and were utilised in all sessions. **Table 1** shows the number and sex of the children recruited at each school.

**Table 1:** Numbers, ages and gender of children recruited for skin prick testing.

		Kippa Ring	Boondall	Slacks Creek	Maleny	All schools
n		128	124	34	94	380
Sex:M,F		64,64	58,66	18,16	40,54	180,200
Age	All mean age in years (range)	10.7 (9–13)	10.8 (9–13)	10.8 (9–13)	10.7 (9–14)	10.8 (9–14)
	Males mean age in years	10.8	10.8	10.6	10.6	10.7
	Females mean age in years	10.6	10.8	11.0	10.8	10.8

All volunteers were accepted and assessed by the attendant respiratory physician and spirometry recorded. Asthma status was allocated on the basis of short interview and medical examination, their spirometry result and their ISAAC questionnaire. All children with a doctor’s diagnosis of asthma were accepted as such. After SPT, children with asthma in the previous 12 months were classified as the ‘asthma’ (n = 97) group and children who had no allergic or respiratory symptoms at all were classified as ‘healthy’ (n = 107). The ‘asthma’ group included children with asthma of varying severity based upon the number of asthma episodes in the last 12 months: ‘mild’, 1 to 3; ‘moderate’, 4 to12; and ‘severe’, more than 12. Others were designated ‘dry cough’ (dry cough only), ‘atopic’ (hay fever and eczema symptoms only) and ‘inactive asthma’ (asthma not in last 12 months). No further analysis of these last three groups features here. Health status groups are shown in **Table 2**.

**Table 2:** Numbers of children in each health status group as determined by responses to ISAAC information.

	Healthy	Asthma			Dry cough	Atopic	Inactive asthma	Total (Males, Females)
		Mild	Moderate	Severe				
Kippa Ring	33	19	7	5	10	17	37	128(64,64)
Boondall	29	22	9	3	14	21	26	124(58,66)
Slacks Creek	10	8	2	1	0	6	7	34(18,16)
Maleny	35	8	11	2	7	20	11	94(40,54)
		57	29	11				
Total	107	97	31	64	81	380	Total	107

### Allergen testing

All children were skin prick tested with commercial allergens in class groups according to names in alphabetical order, with a positive histamine and negative saline control. Allergens were dropped onto a grid drawn on the volar forearm, skin pricked with a lancetter in the middle of the allergen droplet. The resultant wheals were compared to controls and the diameter measured after 10 minutes.

Allergens were obtained from Bayer Corporation, Sydney. *Melaleuca* and *Ligustrum* (privet) were requested but were not available. Allergens used were (as shown on the label of each):Standardized mite *Dermatophagoides farina;* Cockroach Mix 6585 (American, German); Standardized cat pelt; Dog hair—dander; *Hormodendrum cladosporioides; Alternaria tenuis;* Johnson grass, *Sorghum halepense;* Bahia grass, *Paspalum notatum;* Ryegrass, perennial, *Lolium perenne;* Bermuda grass, *Cynodon dactylon;* Olive, *Olea europea;* Acacia, Golden, *Acacia longifolia;* Ragweed, mix GS (giant short); Pine, Aus/SW; She-oak, *Casuarina glauca; and* Eucalyptus/blue gum, *Eucalyptus globulus*.

### Measures

For all SPT measures, a positive result was wheal diameter 3mm or greater that formed after 10 minutes from skin prick. Irregular shaped wheals were calculated by taking the width and adding the length and dividing by two.

### Statistics

Calculations were performed using SPSS Version 22. Charts were produced using Microsoft Excel.

## Results

### Schools groups comparisons

**Fig 7:**
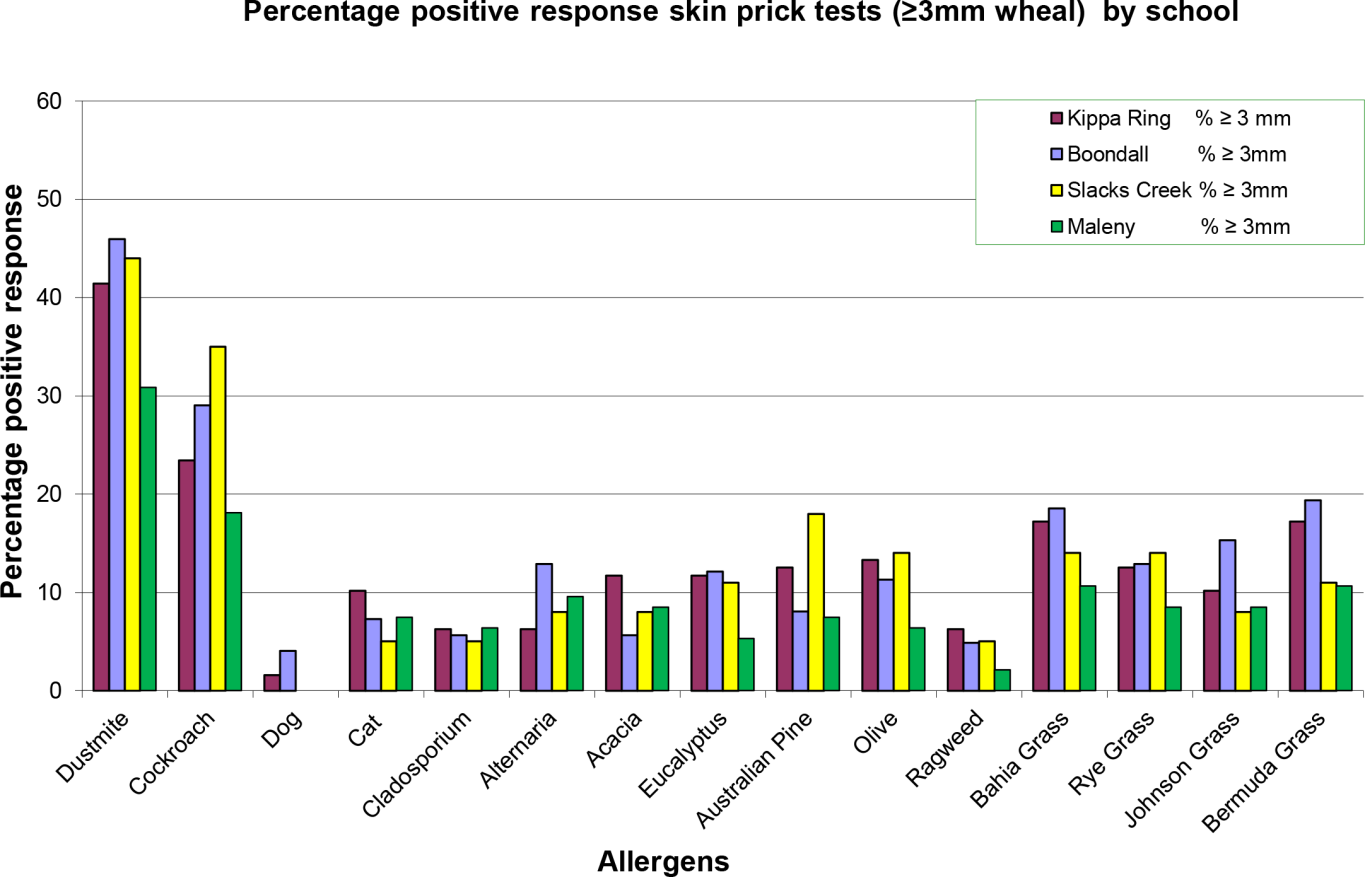
Percentage positive SPT response (≥3mm wheal) in children by school.

Each school group of children with and without symptoms was compared against each other school. For the four schools, the percentage of positive responses (wheals ≥3mm) for ‘dust mite’ (30.9%M-46%B), ‘cockroach’ (18.1% M-35%SC), ‘Bermuda grass’ (10.6%M-19.4%B) and ‘Bahia grass’ (10.6%M-18.5%B) were highest, and responses for ‘dog’ (1.6%KR-4%B), the lowest. All schools SPT response rates were most uniform for ‘*Cladosporium’* (5%SC-6.4%M). These can be seen charted in Fig 7.

### Coastal versus range school comparisons

For the range and coast comparisons, three coastal schools were compared, as a group, with Maleny participants. Fisher’s Exact Test applied to range and coastal SPT positive (wheals≥3mm) responses revealed that dust mite, *Eucalyptus* and cockroach allergen responses, respectively, significantly distinguished coastal from range scores as shown in Table 3 (α set at .05).

**Table 3:** Coast and range comparisons: SPTs ≥3mm wheals from all children of all symptom groups combined applying Fisher’s Exact Test.

	Coast group (n = 252) percentage ≥3mm wheal	Range group (n = 89) percentage ≥3mm wheal	Exact significance tail	Exact significance tail
Dust mite	43.7%	30.9%	.030	.018
Cockroach	27.3%	18.1%	.076	.047
Dog	2.4%	0.0%	.201	.134
Cat	8.4%	7.4%	1.000	.483
*Cladosporium*	5.9%	6.4%	.808	.522
*Alternaria*	9.4%	9.6%	1.000	.554
Acacia	8.7%	8.5%	1.000	.568
*Eucalyptus*	11.9%	5.3%	.078	.046
Australian Pine	11.2%	7.4%	.335	.202
Olive	12.6%	6.4%	.128	.065
Ragweed	5.6%	2.1%	.262	.135
*Bahia*	17.5%	10.6%	.142	.075
Ryegrass	12.9%	8.5%	.276	.167
Johnson grass	12.2%	8.5%	.452	.214
Bermuda grass	17.5%	10.6%	.142	.075

### Severity

No significant differences in severity between coast and range groups were found (t = 1.41, df 95, p = .16). Asthma severity comparisons between coast and range groups were determined by assigning a weighted variable for ‘mild’(1), ‘moderate’(2) and ‘severe’(3) asthma to each participant in those groups. The measure reflects number of episodes of asthma noted on the participant’s ISAAC questionnaire.

### ‘Healthy’ and ‘asthma’ comparisons

**Fig 8:**
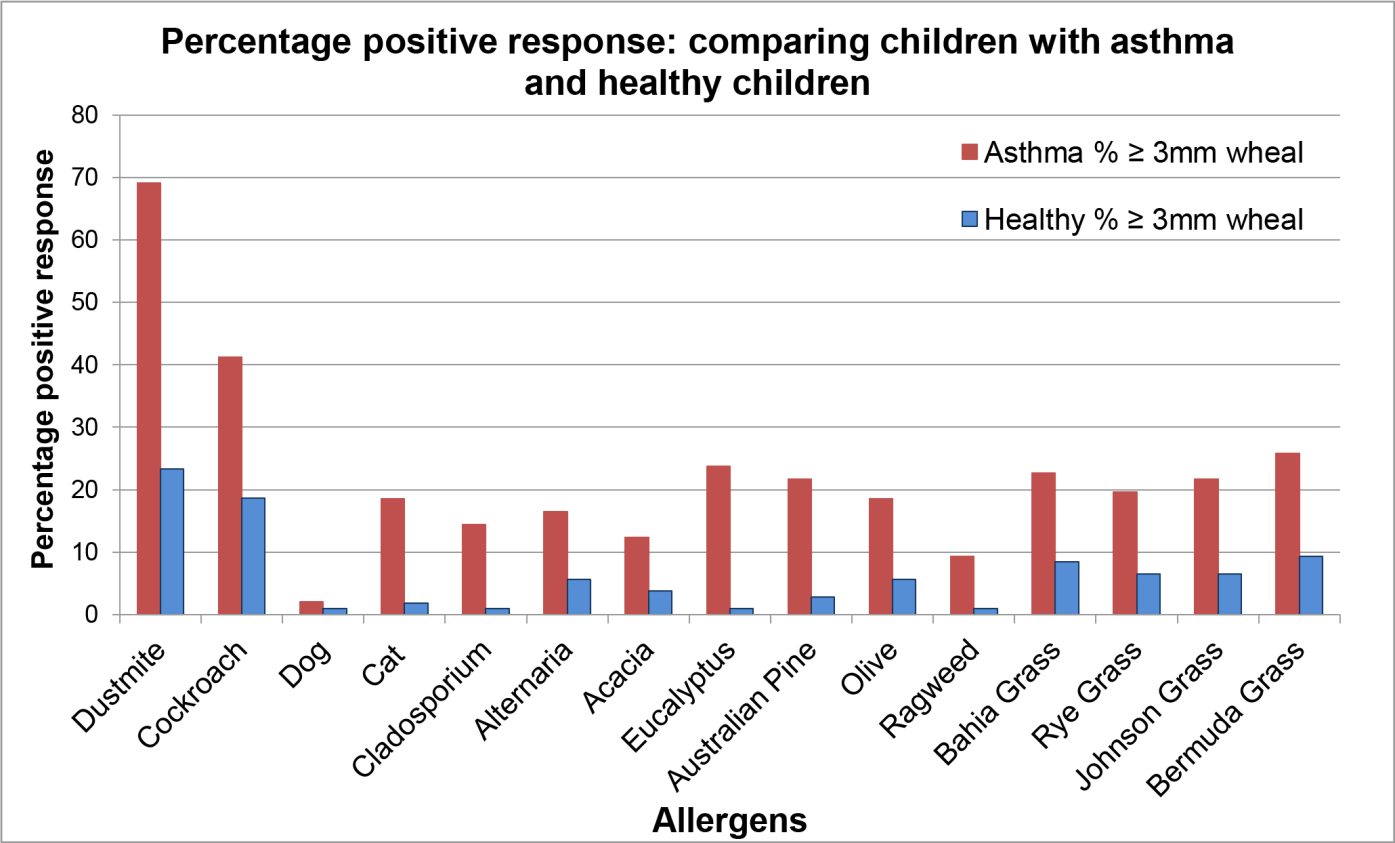
Percentage positive SPT response (*≥3mm wheal)* in children with asthma and healthy children.

Percentage positive response comparisons between groups comprising only ‘healthy’ and ‘asthma’ groups from all schools combined are shown in Table 4 and charted in **Fig 8**. Odds of a response to allergens with wheal size ≥3mm by a child with asthma, compared to a healthy child, are also shown in Table 4.

**Table 4:** Percentage positive SPT response (≥ 3mm wheal) and odds for children with asthma and healthy children.

	Children with asthma(n = 97)	Healthy children(n = 107)		
Allergen	Percentage(count)	Percentage	Allergen	Percentage(count)
Dust mite	68.0%(66)	23.4%(25)	7.0	(3.8–13.0)
Cockroach	40.0%(39)	18.7%(20)	2.9	(1.6–5.5)
Dog	2.1%(2)	0.9%(1)	2.2	(0.2–25.0)
Cat	18.6%(18)	1.9%(2)	12.0	(2.7–53.1)
Cladosporium	14.4%(14)	0.9%(1)	17.9	(2.3–138.7)
Alternaria	16.5%(16)	5.6%(6)	3.3	(1.2–8.9)
Acacia	12.4%(12)	3.7%(4)	3.6	(1.1–11.7)
Eucalyptus	22.7%(22)	0.9%(1)	31.1	(4.1–235.7)
Casuarina	21.6%(21)	2.8%(3)	9.6	(2.8–33.3)
Olive	18.6%(18)	5.6%(6)	3.8	(1.4–10.1)
Ragweed	9.3% (9)	0.9%(1)	10.8	(1.3–87.2)
Bahia grass	22.7%(22)	8.4%(9)	3.2	(1.4–7.3)
Ryegrass	19.6%(19)	6.5%(7)	3.5	(1.4–8.7)
Johnson grass	21.6%(21)	6.5%(7)	3.9	(1.6–9.8)
Bermuda grass	25.8%(25)	9.3%(10)	3.4	(1.5–7.6)

*The odds of obtaining ≥3mm wheal diameter SPT response to an allergen in children with asthma compared healthy children e.g. children with asthma are 31.1 times more likely to be allergic to *Eucalyptus* and 17.9 times more likely to be allergic to *Cladosporium* compared to children with no symptoms

Positive response was greatest for dust mite, cockroach mix, and Bermuda grass for both groups respectively. The children of the ‘asthma’ group demonstrated a higher positive response to all allergens compared to the ‘healthy’ group.

For the ‘asthma’ group, of the plant allergens, Bermuda grass (26%) exceeded *Eucalyptus* (24%) and Bahia grass (23%) followed by Australian Pine (*Casuarina*) (22%), Johnson grass (22%) and ryegrass (20%). Cat (19%) and *Alternaria* (17%) featured among animal and fungal responses.

The allergen response that resulted in the highest odds ratio (Table 4) was *Eucalyptus* (OR 31) followed by *Cladosporium* (OR 18). Thus the odds of a SPT response to *Eucalyptus*, greater than 3mm wheal, was 31 times more for a child with asthma than for a child with no symptoms of allergy or asthma. A post-hoc analysis of *Eucalyptus* allergen responses (two-way Anova) revealed no significant differences between males or females (F = .052; p = .857). There was no significant interaction between ‘group’ (asthma or healthy) and ‘sex’ (male or female) (F = .029; p = .864). *Eucalyptus* scores for asthma or healthy groups differed significantly (F = 1586; p = .016). Background data for Table 4 is shown in Table 5. Despite a significant sex difference in group membership, the impact of sex upon *Eucalyptus* scores is not significant.

**Table 5:** Background data for Table 4.

		Children with asthma	Healthy children	P value
Age	Mean in years, n	10.7	10.9	
	T Test:t = -1.616			.108
Sex	Females %, n	38%,41	62%,68	
	Males%,n	59%,56	41%,39	
	Pearson Chi Sq = 9.62			.002
Comorbidity	Allergic rhinitis%,n	74%,72	Symptom free	
	Atopic dermatitis %,n	41%, 41	Symptom free	

## Discussion

The findings support the hypothesis that nearby vegetation may be related to an increased SPT response to those plants commonly found in coastal SE Queensland.

Response to *Eucalyptus* allergen significantly differed when comparing Maleny to the three coastal schools together (Table 3). This may be due to the large tracts of Myrtaceae family, trees on the coast.

The substantial SPT response is despite the use of commercial allergen *Eucalyptus globulus*, or Tasmanian Blue Gum, found naturally in Tasmania, 2000 km away[20], not in S.E.Queensland. SPT response to *Eucalyptus* does not appear to be species specific perhaps because the volatiles emitted from foliage of local *Eucalyptus tereticornis* are similar[21]. A possible explanation for the phenomenon comes from Gonzalez et al [22] in their explanation of how people sensitized to olive pollen can react to unrelated plant pollens. They conclude that allergic reactions to many different sources can be attributable to a few cross-reactive components.

Cross-reactive responses may also explain the substantial response to olive allergen here. Olive growth in Brisbane is restricted to the occasional garden specimen which typically does not fruit. Olive allergen was included in the test battery as a surrogate for privet, a weed on the range area. Neither privet nor olive is present in substantial numbers on the coast. Cross reactivity of olive has been established with privet, pine, birch, mugwort and cypress [22]. Plants from completely different families, genera and species can be cross-reactive. Extensive plantation pine forests that lie between northern Brisbane and Maleny could be relevant in explaining the substantial response in all schools. As well, or alternately, the response may be linked to common volatile terpenoids like linalool which occurs in olive[23], privet[19], *Melaleuca*[18] and *Eucalyptus*[24] species. Terpene enzymes[25], such as linalool synthase [26], 3-carene synthase [27] and could play a role in cross-reactive allergen responses. Molecular weights of these enzymes are within range of IgE binding components identified in *Melaleuca*[28]^.^

Proximity to highways and increased traffic could result in higher response rates for many allergens for the coastal schools compared to Maleny. This is consistent with views by Behrendt et al. [29] however responses here to *Acacia*, *Alternaria* and *Cladosporium* do not fit this explanation. Some responses are lower than those of the Maleny group.

The olive result is a reminder that the responses to particular plant allergens must be interpreted carefully. The response to olive on the coast, despite is apparent absence, is similar to that for Hirschwehr et al [30] in their description of allergic responses to ragweed allergen in the absence of likely sensitization to ragweed in Germany. They note that ragweed is rare in Germany. This study highlights the problems due to local responses and cross-reactivity inherent in the interpretation of SPT results.

Results here invite investigation into chemical similarities to other plants associated with asthma and allergies. Given the role of cross-reactions in responses to SPT with plant allergens, is the substantial response to Bahia grass in this study because of its demonstrated cross-reactivity with *Melaleuca*[28]?

The key to the cross-reactivity door may lie in the organic compounds that make up the volatile organic emissions of plants world-wide. The volatile emissions from *Eucalyptus* [31]are also found in other species [32–36] associated with allergy and asthma. *Eucalyptus*, native to Australia, has been planted in U.S.A. since the 1860s and extensive plantations can be found in India, Brazil, China, Africa, Spain, Portugal and Pakistan [37]. It may be time to explore the contribution of floral volatiles from *Eucalyptus* and other members of the Myrtaceae family, especially *Melaleuca*, to asthma and allergic response.

Results supported the hypothesis that pollen allergen response from Eucalyptus flowers would differ between coastal and range schools. As well, cross-reactivity of a local plant with olive species, not growing in the area is likely. Analysis of chemical compositions would suggest *Melaleuca quinquenervia* and/or *Eucalyptus* species, because of common terpenoid components. Appreciation for the complexity of chemical relationships among different species and genera can improve understanding of national and international patterns of allergic response to plants.
